# A unique bleeding-related complication of sorafenib, a tyrosine kinase inhibitor, in advanced hepatocellular carcinoma: a case report

**DOI:** 10.1186/1752-1947-8-72

**Published:** 2014-02-26

**Authors:** Ha Yan Kang, Sung Hoon Moon, Il Han Song

**Affiliations:** 1 Division of Hepatology, Department of Internal Medicine, Dankook University College of Medicine, 16-5 Anseo-dong, Cheonan 330-715, Chungnam, Republic of Korea; 2 Department of Internal Medicine, Hallym University Sacred Heart Hospital, 896 Pyeongchon-dong, Anyang 431-070, Gyeonggi-do, Republic of Korea

**Keywords:** Hepatocellular carcinoma, Sorafenib, Tyrosine kinase inhibitor, Hemobilia, Complication

## Abstract

**Introduction:**

Sorafenib, a multikinase inhibitor as a standard of care for advanced hepatocellular carcinoma, may lead endothelial cells to an unstable state by blocking the signaling pathway of vascular endothelial growth factor receptor, which may result in the disruption of the architecture and integrity of the microvasculature, and eventually increase the risk of hemorrhage. Hemobilia is a relatively uncommon condition as a consequence of hepatocellular carcinoma and its risk factors remain uncertain.

**Case presentation:**

Here we report a unique case of hemobilia occurring in a 55-year-old Korean man with hepatitis B virus-related hepatocellular carcinoma on Barcelona Clinic Liver Cancer advanced stage after seven days of treatment with sorafenib. He had received prior radiation therapy. Endoscopy revealed bleeding from the major duodenal papilla and endoscopic retrograde cholangiography revealed an amorphous filling defect throughout the common bile duct. Blood clots were removed by balloon sweeping and a nasobiliary drainage tube was placed. No further bleeding has been detected as of eight months after discontinuation of sorafenib.

**Conclusion:**

Sorafenib may increase the risk of biliary bleeding in hepatocellular carcinoma patients who were primed with irradiation, by blocking the signaling pathway of the vascular endothelial growth factor receptor. Therefore, sorafenib should be used with caution in patients with advanced hepatocellular carcinoma, especially when combined with radiation therapy.

## Introduction

Hemobilia is a very rare manifestation of hepatocellular carcinoma (HCC) [1]. Almost all cases occur in patients who have accompanying bile duct invasion [2,3]; however, the predisposing factors in these patients are uncertain. Sorafenib, a multikinase inhibitor, is recognized as a standard of care for advanced HCC accompanied by extrahepatic spreading or macrovascular invasion. Recently, it was thought that sorafenib can increase the risk of hemorrhage by inhibiting a signaling pathway of the vascular endothelial growth factor (VEGF) receptor [4]. Bleeding events, which can be caused by anti-VEGF signaling therapy, such as sorafenib, sunitinib or bevacizumab, include epistaxis, hemoptysis, gastrointestinal bleeding, vaginal bleeding and brain hemorrhage [5]. Epistaxis is the most commonly reported bleeding episode which is self-limited to dose adjustment; however, gastrointestinal bleedings inclusive of pulmonary or intracranial hemorrhage are reported relatively less often but may be fatal [4,6]. Here, we report a case of hemobilia occurring in a patient with advanced HCC after administration of sorafenib, with a review of the literature.

## Case presentation

A 55-year-old Korean man with HCC associated with liver cirrhosis secondary to chronic hepatitis B virus infection was admitted because of hematemesis and epigastric pain. He had undergone five sessions of transhepatic arterial chemoembolization therapy in the past 12 months. Three weeks previously, he had received radiation therapy due to bone metastasis at the level of the 11th thoracic vertebra with spinal cord compression and then, one week previously, sorafenib was started at a dose of 400mg twice daily. At that time, our patient’s Child-Pugh’s classification was A and Eastern Cooperative Oncology Group performance score was 2.

On admission, his blood pressure was 120/80mmHg and pulse rate was 74 beats per minute. Digital rectal examination was negative and a small amount of fresh blood was drained via a nasogastric tube. Laboratory data included the following: hemoglobin 15.3g/dL, platelet 26,000/mm^3^, total bilirubin 12.2mg/dL, albumin 4.1g/dL, aspartate aminotransferase 192U/L, alanine aminotransferase 181U/L, alkaline phosphatase 301IU/L, gamma glutamyltransferase 137U/L, amylase 58U/L, lipase 26U/L and a prothrombin time of 16.7 seconds. Child-Pugh’s classification was B.

**Figure 1:**
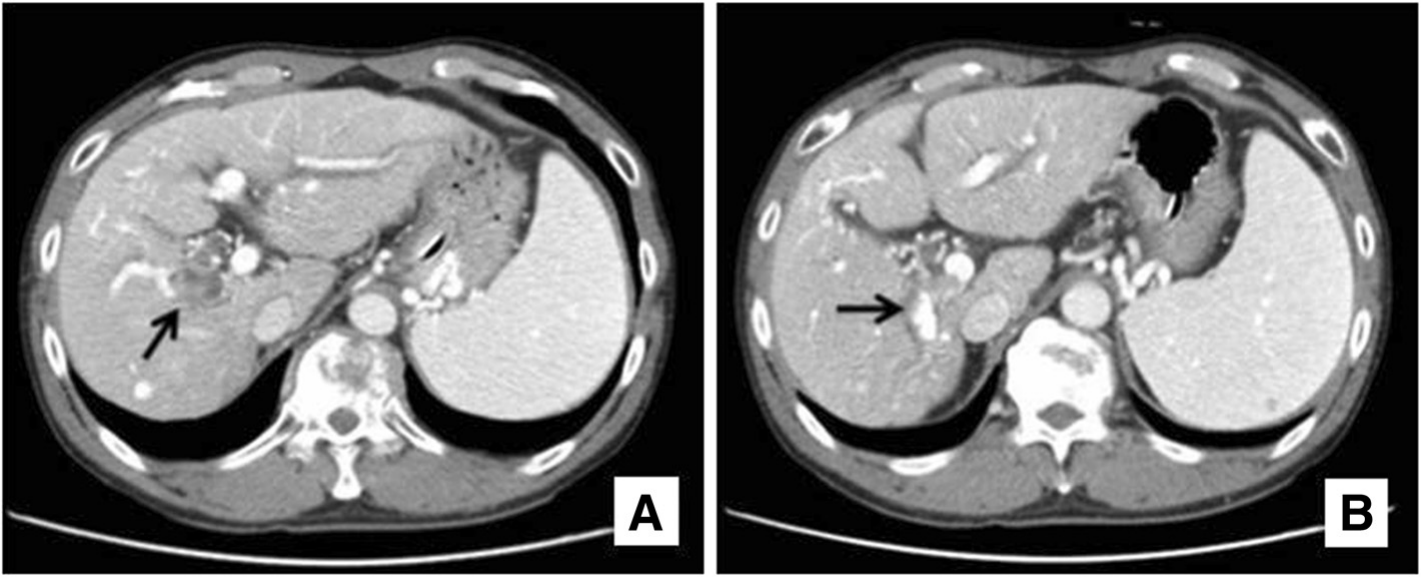
**Computed tomography. (A)** A thrombus was noted in the posterior branch of right portal vein (arrow). **(B)** Dilatation of intrahepatic duct (arrow) adjacent to a partially lipiodolized nodule suggested direct invasion of the tumor into the surrounding intrahepatic duct in the portal-venous phase.

**Figure 2:**
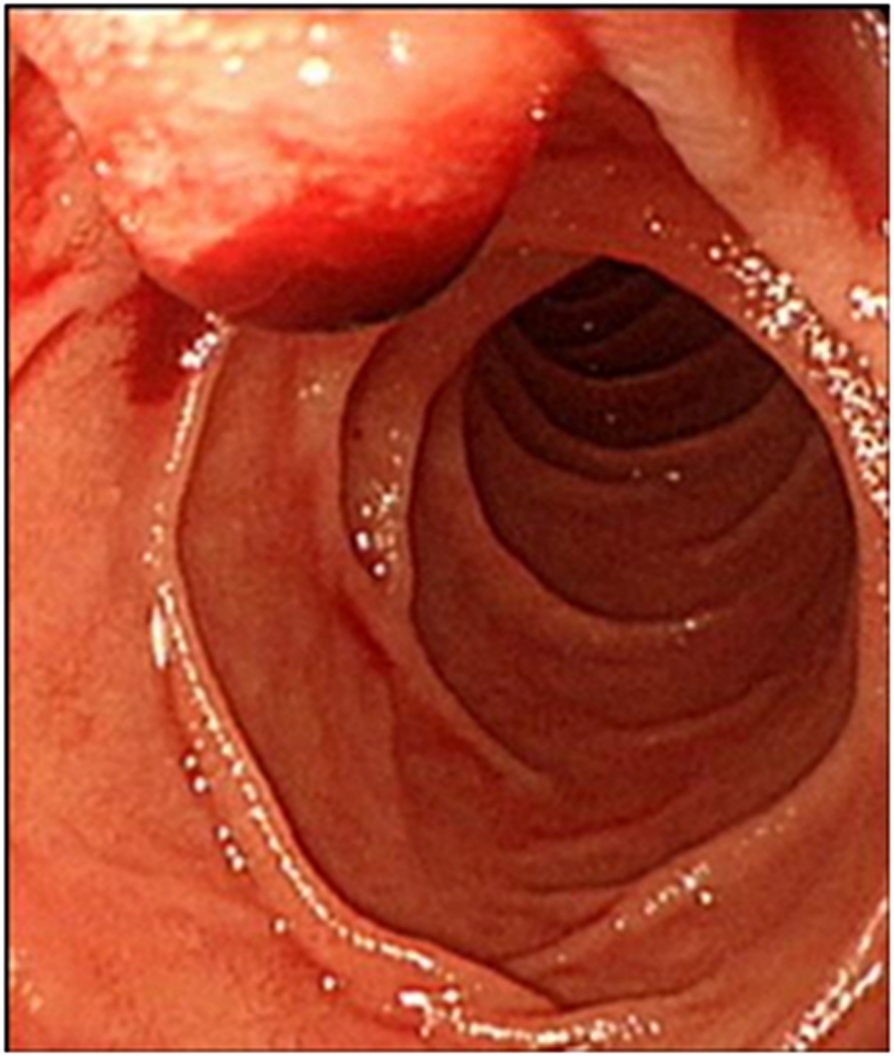
**Upper gastrointestinal endoscopy.** Fresh blood was gushing from major duodenal papilla.

**Figure 3:**
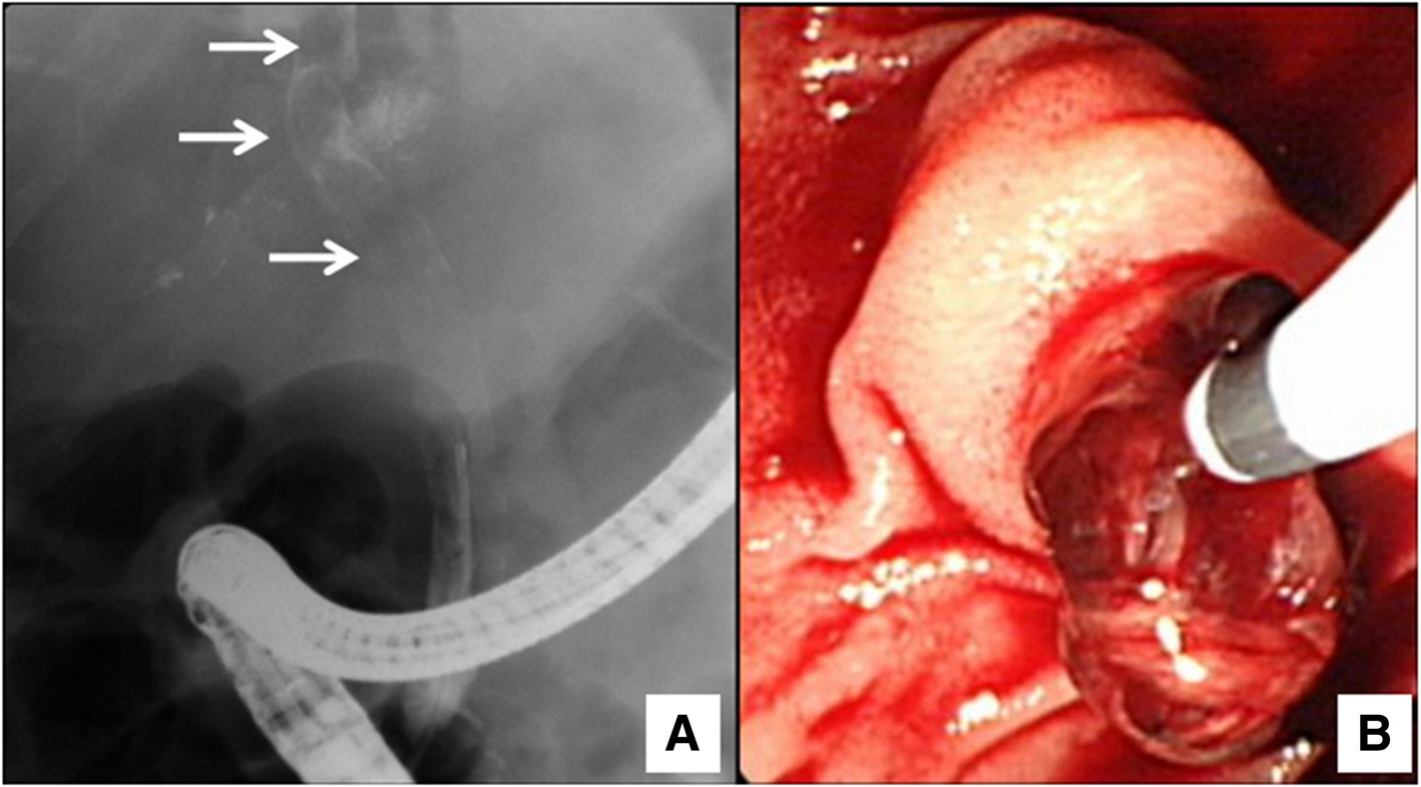
**Endoscopic retrograde cholangiography. (A)** An amorphous filling defect was seen throughout the common bile duct (arrows). **(B)** Blood clots were removed by balloon sweeping.

Abdominal computed tomography (CT) demonstrated a thrombus in the posterior branch of right portal vein (Figure 1A) and dilatation of intrahepatic bile duct (Figure 1B) adjacent to a partially lipiodolized nodule in the portal-venous phase. Upper gastrointestinal endoscopy showed fresh blood emerging from major duodenal papilla (Figure 2). Endoscopic retrograde cholangiography revealed an amorphous filling defect throughout the common bile duct (Figure 3A). Blood clots were removed by balloon sweeping (Figure 3B) and a nasobiliary drainage tube was placed.

After discontinuation of sorafenib administration, with a regular irrigation of saline via nasobiliary tube, our patient became hemodynamically stable and total bilirubin level decreased to 4.6mg/dL. He was discharged and no further bleeding has been detected as of eight months after the episode.

## Discussion

Hemobilia is a relatively uncommon cause of gastrointestinal bleeding and is usually associated with trauma, cholelithiasis, acalculous inflammatory diseases, vascular disorders and tumors. When HCC invades the biliary tree, hemobilia can occur due to rupture of the tumor into the biliary system [2]. In our present case, tumor invasion into the intrahepatic bile duct was suspicious, but not definite on the abdominal imaging scan.

Kojiro *et al.*[3] found hemobilia occurring in 5 (21%) of 24 HCC cases with tumor growth within bile ducts; however, it is unclear how many cases of hemobilia develop in patients with HCC and what the predisposing factors are. Verset *et al.*[7] has reported two cases of fatal hemobilia in patients with advanced HCC on the second and seventh day after sorafenib therapy and our present patient was also admitted with hematemesis on the seventh day after sorafenib administration. Adverse events of VEGF receptor-targeted agents relevant to bleeding have been usually reported to occur early in the course of treatment [8,9]. Sorafenib administration itself may be the potential cause of the development of gastrointestinal bleeding presented as hemobilia in our patient.

Sorafenib is a small molecule inhibitor of multiple tyrosine kinase receptors that regulates cell proliferation and angiogenesis. It is now the standard of care for patients with advanced-stage HCC with macrovascular invasion or extrahepatic spread [5,10]. Rash, exfoliative dermatitis, hand-foot skin reaction, diarrhea, fatigue and hypertension are common adverse effects of sorafenib [4,11]. Although there are still some debates whether sorafenib may increase the risk of bleeding, a recent meta-analysis showed a significant two-times increased risk of a bleeding event in cancer patients treated with sorafenib or sunitinib [12]. These bleeding events can be thought of as being associated with the aberrant disruption of VEGF signaling involved in mechanisms related to vascularization and coagulation. VEGF stimulates endothelial cell proliferation as well as promotes endothelial cell survival, which, consequently, helps maintain the architecture and integrity of the microvasculature [11]. Therefore, inhibition of VEGF signaling can decrease the renewal capacity of the endothelial cell in response to trauma, which causes endothelial dysfunction, and eventually increases the risk of hemorrhage. Furthermore, it is considered that the weakening of the walls of major vessels by tumor erosion, necrosis, cavitation or other concurrent pathological conditions are also likely to play a central role in causing hemorrhage in patients on anti-VEGF therapy [4]. In the present case, abdominal CT performed six weeks prior to the bleeding event showed suspicious tumor invasion into the intrahepatic duct adjacent to the primary nodule; however, at that time, there was no evidence of bleeding radiologically as well as clinically. Hemobilia was found on the seventh day after sorafenib therapy began and the bleeding stopped with conservative management with sorafenib discontinuation. It was thought that the tumor had weakened the wall of the portal vein and sorafenib precipitated bleeding into the bile duct by blocking VEGF signaling.

Hui *et al.*[13] reported that the risk of bleeding with sunitinib, one of the VEGF receptor tyrosine kinase inhibitors, was higher in patients with nasopharyngeal carcinoma who received prior high-dose radiation therapy than those who did not. Irradiation causes damage to the endothelial cells in the capillary network and negatively influences the subsequent vascular repair process [14], and also enhances sensitivity of endothelial cells to anti-VEGF therapy [15]. In the present case, the patient received radiation therapy for two weeks prior to sorafenib administration. Previous radiation therapy may be another factor contributing to increased risk of bleeding in HCC patients on anti-VEGF therapy.

## Conclusions

Sorafenib may increase the risk of bleeding in HCC patients who were primed with irradiation, by disruption of the architecture and integrity of the microvasculature, which may result from blocking of the signaling pathway of the VEGF receptor. Therefore, sorafenib should be used with caution in patients with advanced HCC, especially when combined with radiation therapy.

## Consent

Written informed consent was obtained from the patient for publication of this case report and accompanying images. A copy of the written consent is available for review by the Editor-in-Chief of this journal.

## Abbreviations

CT: Computed tomography; HCC: Hepatocellular carcinoma; VEGF: Vascular endothelial growth factor.

## Competing interests

The authors declare that they have no competing interests.

## Authors’ contributions

KHY drafted the manuscript and was a member of the clinical team. MSH provided an expert endoscopic procedure and obtained endoscopic images. SIH oversaw clinical management and supervised the present case report. All authors read and approved the final manuscript.

## Authors’ information

Ha Yan Kang and Sung Hoon Moon are co-authors.
